# Impact of artificial intelligence on the academic performance of nursing students: a quasi-experimental study at the Higher Institute of Nursing Professions and Health Techniques in Oujda

**DOI:** 10.1192/j.eurpsy.2026.11492

**Published:** 2026-07-31

**Authors:** S. Essadki, S. Bechrouri, N. Ouahbi, F. Wahoud, R. Lamsyah, W. Hajjaji, A. Zarouali, F. Touhami, A. Mouzazi, M. El Baz, M. Benachour, H. Elmsellem, M. A. Lafraxo

**Affiliations:** 1 Higher Institute of Nursing Professions and HealthTechniques; 2 Laboratory of Maternal, Infant, and Mental Health(LSMIM), Faculty of Medicine and Pharmacy, Oujda; 3 Higher Institute of Nursing Professions and HealthTechniques, Fez; 4 Laboratory of Biology and Health, Faculty of Science, Ibn Tofail University, Kenitra, Morocco

## Abstract

**Introduction:**

With the rise of Artificial Intelligence (AI) in higher education, it has become essential to assess its impact on the academic performance of nursing students.

**Objectives:**

This study aims to compare the impact of traditional teaching and an AI-generated video-based method on the academic performance of students at the Higher Institute of Nursing Professions and Health Techniques of Oujda.

**Methods:**

A quasi-experimental study was conducted among 68 polyvalent nursing students enrolled in the fourth semester of the Bachelor’s program. Each participant attended two successive teaching interventions within the module Psychiatric Disorders and Nursing Care: a traditional lecture and a session integrating an educational video produced by AI. For each session, a pre-test and post-test were administered to assess students’ learning outcomes. Paired-sample t-tests were used to compare the mean scores, with a 95% confidence interval (CI 95%) and a significance level set at p = 0.05.

**Results:**

Students showed a significant improvement in their results after both teaching methods. However, the traditional method led to a higher average gain of 10.27 points, compared to 2.60 points with the AI-based method, with a highly significant difference (p < 0.001).

**Conclusions:**

Despite the pedagogical potential of AI, the findings indicate that traditional teaching remains more effective in improving academic performance. The lack of interaction and the uniformity of AI-generated content may explain these limitations. A hybrid integration combining human pedagogical guidance with AI tools appears to be a more suitable approach to optimize learning.

**Disclosure of Interest:**

None Declared

